# Zinc-oxide nanoparticles ameliorated the phytotoxic hazards of cadmium toxicity in maize plants by regulating primary metabolites and antioxidants activity

**DOI:** 10.3389/fpls.2024.1346427

**Published:** 2024-01-18

**Authors:** Mujahid Hussain, Rehana Kaousar, Syed Ijaz Ul Haq, Changfeng Shan, Guobin Wang, Nadia Rafique, Wang Shizhou, Yubin Lan

**Affiliations:** ^1^College of Agricultural Engineering and Food Science, Shandong University of Technology, Zibo, Shandong, China; ^2^National Center for International Collaboration Research on Precision Agricultural Aviation Pesticides Spraying Technology (NPAAC), Ministry of Science and Technology, College of Electronics Engineering, South China Agricultural University, Guangzhou, China; ^3^Department of Biological and Agricultural Engineering, Texas A&M University, College Station, TX, United States

**Keywords:** abiotic stress, nanoparticles, photosynthetic pigments, antioxidants, metabolites

## Abstract

Cadmium stress is a major threat to plant growth and survival worldwide. The current study aims to green synthesis, characterization, and application of zinc-oxide nanoparticles to alleviate cadmium stress in maize (*Zea mays* L.) plants. In this experiment, two cadmium levels (0, 0.6 mM) were applied to check the impact on plant growth attributes, chlorophyll contents, and concentration of various primary metabolites and antioxidants under exogenous treatment of zinc-oxide nanoparticles (25 and 50 mg L^-1^) in maize seedlings. Tissue sampling was made 21 days after the zinc-oxide nanoparticles application. Our results showed that applying cadmium significantly reduced total chlorophyll and carotenoid contents by 52.87% and 23.31% compared to non-stress. In comparison, it was increased by 53.23%, 68.49% and 9.73%, 37.53% with zinc-oxide nanoparticles 25, 50 mg L^-1^ application compared with cadmium stress conditions, respectively. At the same time, proline, superoxide dismutase, peroxidase, catalase, and ascorbate peroxidase contents were enhanced in plants treated with cadmium compared to non-treated plants with no foliar application, while it was increased by 12.99 and 23.09%, 23.52 and 35.12%, 27.53 and 36.43%, 14.19 and 24.46%, 14.64 and 37.68% by applying 25 and 50 mg L^-1^ of zinc-oxide nanoparticles dosages, respectively. In addition, cadmium toxicity also enhanced stress indicators such as malondialdehyde, hydrogen peroxide, and non-enzymatic antioxidants in plant leaves. Overall, the exogenous application of zinc-oxide nanoparticles (25 and 50 mg L^-1^) significantly alleviated cadmium toxicity in maize. It provides the first evidence that zinc-oxide nanoparticles 25 ~ 50 mg L^-1^ can be a candidate agricultural strategy for mitigating cadmium stress in cadmium-polluted soils for safe agriculture practice.

## 1 Introduction

Heavy metals are continuously deposited into the soil through automobiles, factories, and the disposal of detritus. Metal contamination degrades soil physical and chemical properties with decreased physiological and metabolic activities of plants (Zhou et al., 2019; Basit et al., 2023a). Cadmium (Cd) is a trace element that mainly affects physiochemical processes like photosynthesis and plant-water relationships (Paunov et al., 2018). Similarly, Cd reduced the growth, dry biomass, and morphological characteristics of *Ocimum basilicums* (Fattahi et al., 2019) and significantly reduced *Vigna radiata* production (Rehman et al., 2022). Cd stress increases the production of reactive oxygen species (ROS) and changes plants’ antioxidant potential (Yizhu et al., 2020; Hussain et al., 2022a). In plant biology, ROS serves as cell signaling molecules and induces oxidative stress depending on its concentration (Mittler, 2017). In a stressful environment, high ROS production damages proteins, DNA, RNA and causes chlorophyll degradation (Xuebin et al., 2020).

Nanomaterials have significant potential in addressing environmental contamination and have gained a lot of interest because of their putative potential to accelerate nutrient availability in plants (Basit et al., 2022a; Ulhassan et al., 2023). Zinc-oxide nanoparticles (ZnO-NPs) have a diameter of less than 100 nanometers, high catalytic activity, and large surface area relative to their size (Shirini et al., 2013). It has different chemical and physical behaviors depending on various materials or the routes used for the synthesis. ZnO-NPs are being used in agriculture sciences at a higher rate than other manufactured nanoparticles (Basit et al., 2022b). Different amounts of ZnO-NPs were added in three levels (1, 2.5, 5 mg kg^-1^), and the results showed that nanoparticles increased the pH, maintained rice biomass increased by 13~22%, and 25~43% under the medium and high Cd dosage (Zhang et al., 2019). These are low-level Zn fertilizer that improves mung bean and chickpea development and chlorophyll content (Rizwan et al., 2019b). It also enhanced cotton growth and decreased plant oxidative stress (Venkatachalam et al., 2017b). However, phyco-molecule loaded ZnO-NPs improved growth, photosynthesis, lead (Pb), Cd absorption and reduced abiotic stress in *Leucaena leucocephala* seedlings (Venkatachalam et al., 2017a). The accessibility of hazardous metals such as Cd and Pb may be influenced by the interaction between ZnO-NPs and metals. Several plant species subjected to ZnO-NPs have shown increased chlorophyll and carotenoid contents, including *Solanum lycopersicum*, *Coriandrum sativum*, and *Cucumis sativ*us and *Zea mays* (Singh et al., 2016; Pullagurala et al., 2018; Kataria et al., 2019; Salam et al., 2022). Cd concentrations were reduced by the application of ZnO-NPs in wheat and peas (Pandey et al., 2010; Hussain et al., 2018). ZnO-NPs have various detrimental effects on plants and microbes, which include DNA damage, lysosomal instability, ROS creation, and the reduction of oxidative stress caused by the direct penetration and release of Zn^2+^ ions into plant and microbial cells (Sheteiwy et al., 2021). Therefore, the levels of oxygen gas, H_2_O_2_, ionic leakage, and lipid peroxidation were responsible for the beneficial effects on maize plant development and dry matter accumulation (Alharby et al., 2021). It was concluded that heavy metal stressed plants have reduced nitrate reductase activity, improved antioxidant enzymes, and enhanced the accumulation of amino acid, proline, and sugar content which were related to foliar exogenous efficacy of nanoparticles in maize (Iqbal et al., 2018) and wheat (Hussain et al., 2018). Modern technical advancements are being transformed by an innovative area of scientific study (Griffin et al., 2017).

Maize is a multipurpose crop that produces more than a billion metric tons of grain yearly (Zulfiqar et al., 2022). Cd stress hindered maize development, damaged chloroplast tissues, and negatively impacted growth parameters. Hussain et al. (2013) applied various Cd concentrations 21 days after seedling and concluded that plant growth was significantly reduced under the higher Cd concentration (12 mg kg^-1^ sand). Cd-induced oxidative stress enhances malondialdehyde (MDA) production in maize (Cui and Wang, 2006). Researchers have shown that increased MDA is negatively linked with plant growth and development (Ashraf et al., 2010). The inability of the plasma membrane to control the ions across the cell leads to a negatively effect on total chlorophyll, Chl *a*, *b*, leaf area, root and shoot biomass, and carotenoid contents. Consequently, the current study examined the exogenous efficacy of zinc oxide nanoparticles on maize under Cd stress by hypothesizing that a foliar spray of Zn may increase Cd resistance in maize by increasing the antioxidant activities. The objective of this study was to (1) assess whether the application of ZnO-Nps can alleviate the adverse effect of cadmium stress on the growth and physiology; (2) find out the degree of damage induced by oxidative stress in maize and establish whether an antioxidant system is the major cellular component to combat this hazard; (3) elucidate the underlying mechanisms of cadmium tolerance using the information obtained from various determinants.

## 2 Materials and methods

### 2.1 Experimental site and treatments

Research was conducted to investigate the basic biological and chemical processes of maize resistance to Cd stress. The study was performed at the research area of Shandong University Technology, Shandong. A maize cultivar Deng hai-605 (Shandong Denghai Seeds Co., Ltd.), was used in this trial. Ten seeds were grown in pots, and the soil was sandy loam. Two cadmium chloride (CdCl_2_) levels, including control (0 mM and 0.6 mM), were supplemented with Hoagland’s solution at the seedling stage. Two solutions were made with 25 and 50 mg L^-1^ of zinc oxide nanoparticles. 0.01% Tween-20 was mixed with distilled water to make two bottles of 500 mL volume, and then two dosages of ZnO-NPs (25, 50 mg L^-1^) were mixed separately. These solutions were poured into two separate hand-pressure spray pumps. Firstly, 25 mg L^-1^ ZnO-NPs solution was applied on controlled and Cd stress treatments, and then 50 mg L^-1^ was applied in the same way. One No ZnO-NPs treatment was kept as a control treatment in which no ZnO-NPs were added, but only distilled water was sprayed as an equal amount of water used in ZnO-NPs treatments. The experimental design was (CRD) with four replications. Tissue sampling was made 21 days after treatments to determine the various attributes at the seedling stage.

### 2.2 Preparation of ZnO-Nps

The co-precipitation technique by Devi and Velu (2016) for forming pure ZnO-NPs was employed due to its ability to trace the heavy metal ions and is relatively economical. 0.1 M Zinc nitrate solution was mixed into 100 mL of deionized water while vigorously stirring for 20 minutes to ensure homogeneity. Then, 6 g of sodium hydroxide (NaOH) was added dropwise to the deionized water solution and stirred at 60°C for 3 hours. The final product pH was maintained at the level of pH=10-11. At pH=11, ZnO-NPs were visualized as precipitation on the bottom of the beaker and then washed with deionized water and ethanol in multiple steps to make it free from impurities. At 1792 g, the solution combination was centrifuged and dried over by maintaining the temperature of 60°C for 6 hours. With proper grinding, the desired morphology of ZnO-NPs was formed and characterized by X-ray powder diffraction examination of the materials using Cu, K1 radiation on a Rigaku diffractometer (λ = 1.5406 Å). Additionally, ZnO-NP size was determined using SEM (JSM5910, JEOL).

### 2.3 Growth analysis

Plant materials were collected and properly cleaned. After separating plant parts, root and shoot fresh weight (FW) was taken immediately after uprooting and oven-dried for seven days at 70°C to measure dry weight (DW). The leaf length and width were measured to estimate the leaf area (cm) by the following formula;


L. A=(Length×Width×0.68)

Where 0.68 is the correction factor.

### 2.4 Chlorophyll and carotenoid contents

Fresh plant material was homogenized in 80% methanol and stirred for 15 minutes at 16128 g using the Arnon (1949) technique. Photosynthetic pigments were investigated using the supernatant ultraviolet-visible spectrometer (Hitachi U1800; Tokyo, Japan). The readings were taken at 480, 663, and 645 nm for chlorophyll *a*, *b*, and carotenoids.

### 2.5 Determination of metabolites

#### 2.5.1 Malondialdehyde content

For the MDA determination, 0.5 g leaf samples were grounded in 6% trichloroacetic acid (TCA) and centrifuged for 15 minutes. The readings were noted at 532 and 600nm (Dhindsa et al., 1981).

#### 2.5.2 Hydrogen peroxide content

The H_2_O_2_ concentration was calculated using the Velikova et al. (2000) technique. For analysis, 0.5 g of plant material was grounded with (2 mM) TCA in a mortar placed on an ice bath, stirred at 16128 g, and then 10 Mm of potassium iodide (KI) buffer was mixed with 0.5 mL of the extract. Readings were determined at 390 nm.

#### 2.5.3 Ascorbic acid content

Ascorbic acid was governed by Mukherjee and Choudhuri (1983). Firstly, 0.5 g leaf specimens were grounded with 10% TCA in the ice bath and stirred at 16128 g. However, optical density was determined at 530 nm in a spectrophotometer.

#### 2.5.4 Total phenolic content

Total phenolic content was estimated by Wolfe et al. (2003) method. A 0.25 g extract was grounded in 5 mL of methanol (80%) and stirred for 15 minutes at 16128 g. After that, 0.5 mL of Folin Ciocalteus substance was added to 1.0 mL supernatant extract. Later on, 2.5 mL of Na_2_CO_3_ was added, and 10% volume was formulated using distilled water and cooled for thirty minutes. The readings were noted at 750 nm by using a scale.

#### 2.5.5 Total flavonoid content

Flavonoid content was estimated through the colorimetric test (Ordonez et al., 2006). Firstly, 0.25 grams of leaf sample were grounded in 5 mL methanol and centrifuged at 16128 g. 1 mL extract was assorted with 0.3 mL of Al_2_Cl_3_ and NaNO_3_ by adding 0.2 mL of NaOH. The mixture was mixed up, and the readings were noted at 510 nm with the help of a spectrophotometer using water as a blank.

#### 2.5.6 Anthocyanin contents

Hodges and Nozzolillo (1996) method was used to determine the anthocyanins. In this process, 0.1 g of leaf material was ground in 2 mL of methanol and heated the mixture for 1 hour. After centrifugation, the wavelength was checked at 600 nm by using a spectrophotometer.

#### 2.5.7 Proline contents

For proline determination, Bates et al. (1973) procedure was used. 0.5 g of plant tissue was ground in 10 mL of sulphosalicylic acid and centrifuged. Ninhydrin (1 mL) and glacial acetic acid (2 mL) were inserted and heated. Then, 2 mL of toluene was added for proline extraction. The absorption of the extract was estimated at 520 nm from the spectrophotometer.

#### 2.5.8 Total free amino acid contents

Total free amino acid contents were estimated by Hamilton et al. (1943) technique. Leaf extract of l mL was transferred into test tubes by adding 2% pyridine and 10% ninhydrin solution. Then, water bath the solution at 100°C for thirty minutes. After that, add distilled water to maintain the volume 25 mL in test tubes. Readings were noticed at 550 nm wavelength.

#### 2.5.9 Total soluble proteins

Bradford (1976) technique was used for total soluble protein determination. Firstly, 0.25 g leaf material was grounded in 5 mL phosphate buffer, then centrifuged at 16128 g by adding 2 mL of Bradford reagent and left for thirty minutes at normal temperature. Then, readings were taken at 595 nm spectrophotometrically (Hitachi U-2910, Tokyo, Japan).

#### 2.5.10 Total soluble sugar

Riazi et al. (1985) technique was used to determine total soluble sugar. For this, 0.1 mL of extract was assorted with 2 mL of anthrone substance, and then the subsequent solution was left for thirty minutes. Readings were recorded at 620 nm.

#### 2.5.11 Reducing sugar contents

Plant extract (0.25 g) was ground in 5 mL methanol and centrifuged at 16128 g. A mixture of 0.5 mL plant sample, 1 mL of distilled water, and dinitrosalicylic acid (DNSA) was used to prepare the solution. The readings were checked at 540 nm from the spectrophotometer after a water bath for 15 minutes.

#### 2.5.12 Antioxidants enzymes

Fresh Leaf extract (500 mg) was homogenized in phosphate buffer (10 mL) with a maintained pH of 7.8 and then vortexed the mixture at 16128 g for fifteen minutes at 4°C temperature. After that, it was cooled to determine enzymatic activities (APX, SOD, CAT, and POD). The SOD was estimated by following Giannopolitis and Ries (1977) method. A solution was prepared by mixing 50 µL of enzyme extract, 500 µL of PCR binding solution (SPB), 100 µL of methionine, 500 µL of distilled water, and 50 µL of nitroblue tetrazolium chloride (NBT). It was left inside the cuvette under a lamp for twenty minutes, and readings were taken at 560 nm. CAT and POD activities were calculated by following Chance and Maehly (1955). The CAT reaction mixture contained about 3 mL of potassium phosphate buffer, 0.1 mL of extract, and H_2_O_2_ in the cuvette. The readings were calculated at 240 nm every 20 s using a spectrophotometer. For POD activity plant extract, 0.1 mL was inserted into 1.5 mL of 5 Mm guaiacol and 1.5 mL of H_2_O_2_. The absorbance was taken at 470 nm. A 0.01-unit min^-1^ change in absorbance was considered one unit of CAT and POD activity. The enzyme’s specific activity was measured in enzyme units per mg of protein. Ascorbate peroxidase (APX) was determined using the Krivosheeva et al. (1996) technique. The solution mixture comprised 700 µL lead (Pb), 0.5 Mm ascorbate, 1.5 µL H_2_O_2_, and enzyme extract. Then, the absorbance was measured at 290 nm in decline order at 120 times scan for every 20s.

### 2.6 Statistical analysis

SPSS.10 (SPSS, Chicago, IL, USA) was used for the statistical analysis to determine the mean comparison, difference and interaction between all the treatments at a probability level< 0.05, followed by three-way ANOVA. Origin 2021 software (OriginLab Co., Northampton, MA, USA) was used for fitting all the equations.

## 3 Results

### 3.1 Nanoparticle characterization

#### 3.1.1 X-Ray diffraction analysis

**Figure 1:**
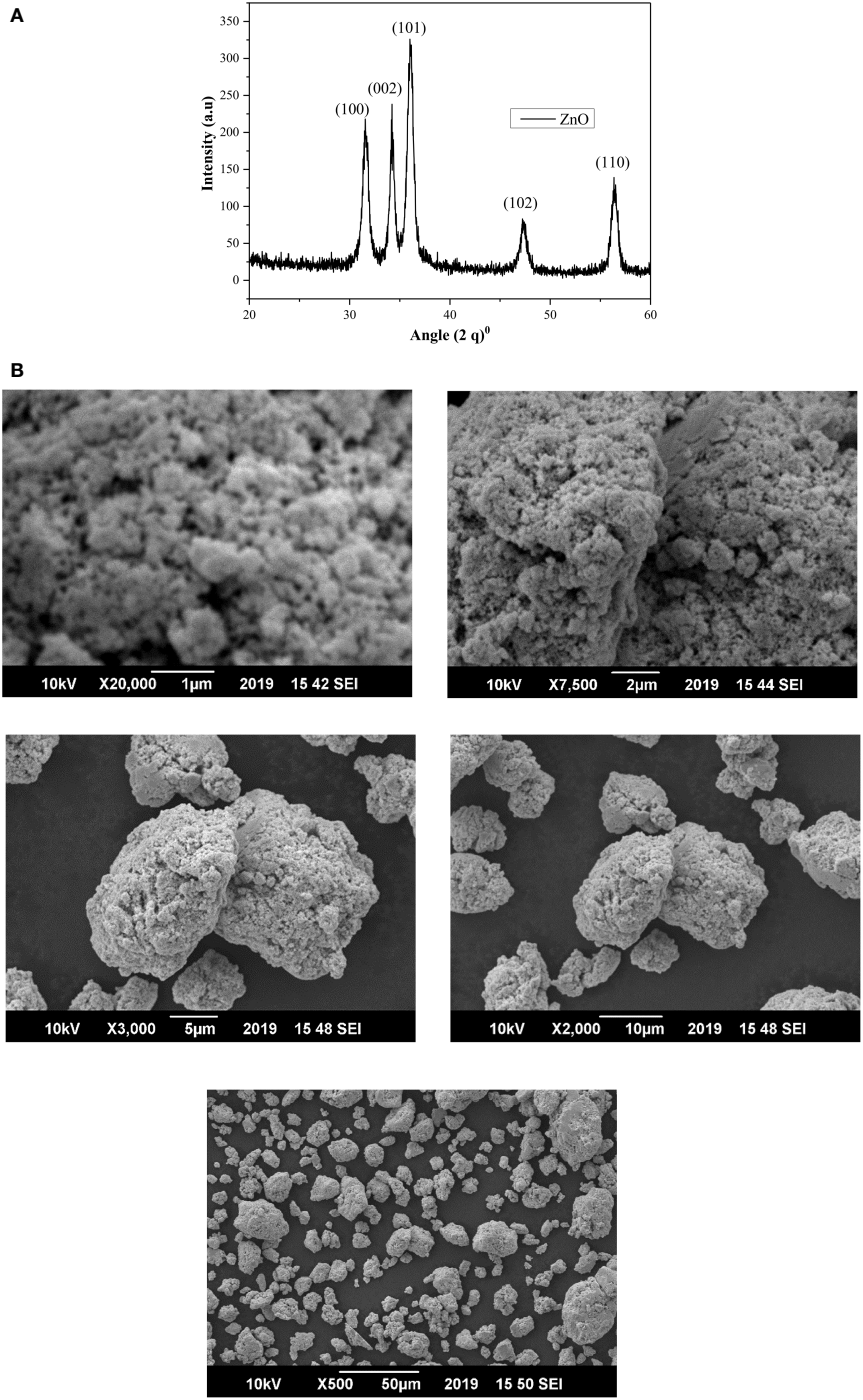
**(A)** The XRD patterns of ZnO-NPs synthesized by the co-precipitation technique. **(B)** SEM images of pure ZnO-NPs at different magnification ranges.

Scanning electron microscope (SEM) and (XRD) pattern images of the ZnO-NPs are shown in **Figures 1A, B**. The X-ray diffraction pattern of ZnO-NPs demonstrated that prepared ZnO-NPs were crystalline. The peaks at 2θ = 31.54°, 34.16°, 36.14°, 47.29°, and 56.41° were assigned [100], [002], [101], [102], and [110] reflection lines of ZnO-NPs material, respectively (**Table 1**). By comparing with XRD spectra of ZnO JCPDS 36-1451, newly synthesized ZnO-NPS depicted the hexagon structure of the clear phase of ZnO crystal. The morphology of the grown ZnO-NPs was determined by SEM image. The structure of the ZnO-NPs with diameters ranging from 70 to 100 nm was shown by the SEM image of ZnO-NPs. Close inspection indicates that these atoms are aggregates of much smaller nanoparticles.

**Table 1:** Effect of foliar ZnO-NPs XRD data of pure ZnO-NPs.

Synthesis parameter	Phases	2(*θ*) Obs	Hkl	FWHM	D-spacing (nm)	R-Intensity	C.S (nm)	Strain (ϵ)	δ × ^−3^ (nm)^-2^
X=0	ZnO	31.54	[100]	0.315	2.837	64.81	24.27	0.0757	1.69
		34.16	[002]	0.236	2.625	56.55	32.14	0.0564	0.97
		36.14	[101]	0.629	2.486	100	11.98	0.1496	6.96
		47.29	[102]	0.551	1.922	21.25	13.20	0.1261	5.74
		56.41	[110]	0.960	1.629	37.08	7.29	0.2115	18.82

### 3.2 Growth attributes

**Figure 2:**
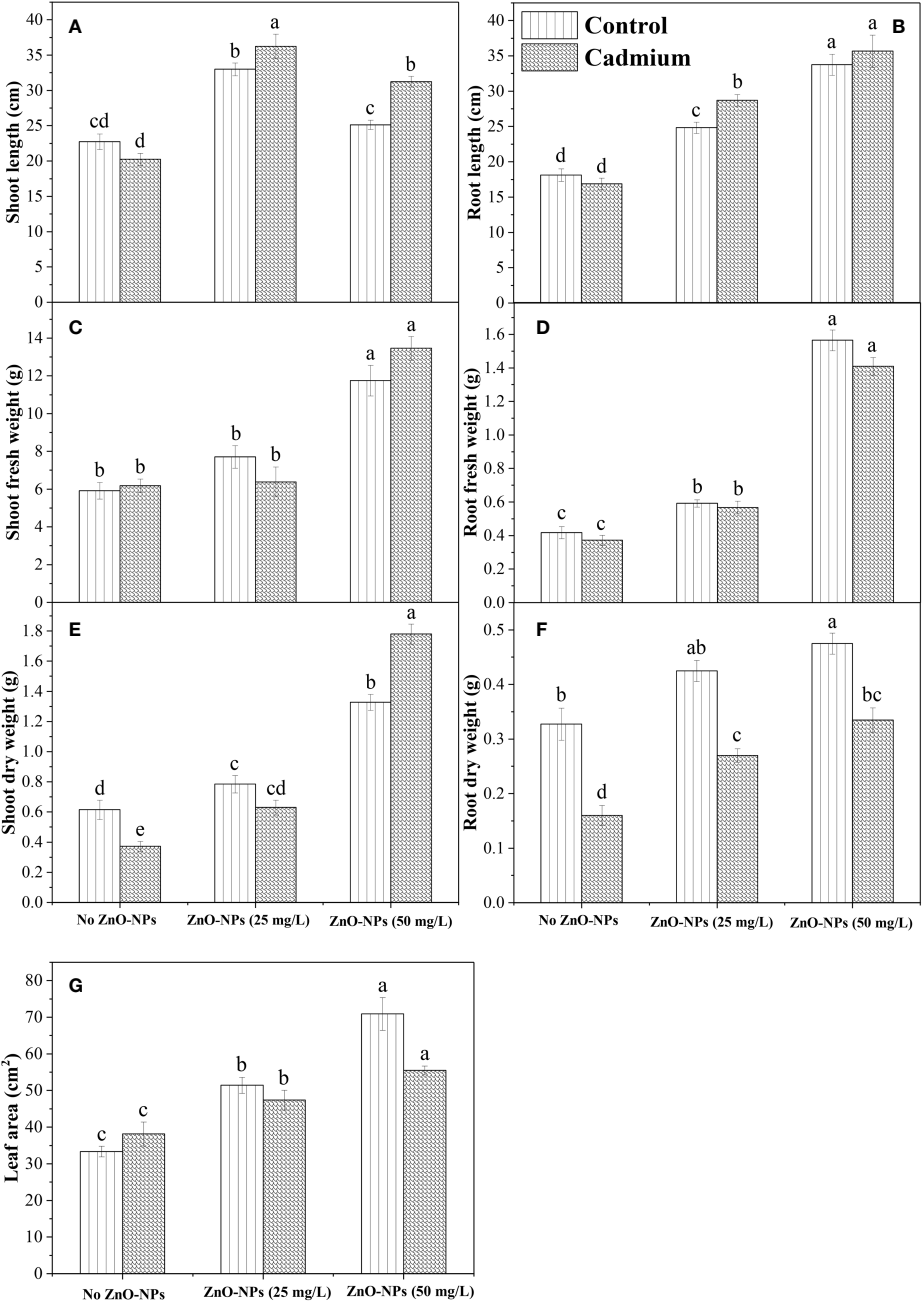
ZnO-NPs influence on morphological attributes of maize plant at vegetative stage under cadmium stress and controlled conditions **(A–G)**. Three-way ANOVA was performed to evaluate the results. Bars with lowercase letters are statistically different at p< 0.05 by the LSD test.

Shoot and root length was significantly changed by ZnO-NPs application under the control and Cd stress compared with no application of ZnO-NPs, while an insignificant difference was observed between control and Cd stress for root and shoot length when no ZnO-NPs were applied. Shoot length and root length were found to be higher under the Cd stress with 25 mg L^-1^ and 50 mg L^-1^ of ZnO-NPS, respectively (**Figures 2A, B**). The interaction between two factors for shoot length was significant, while for root length it was non-significant. For shoot and root fresh weight, no significant difference was observed between the Cd and control with or without ZnO-NPs various dosages. While the ZnO-NPs dosages significantly increased the shoot and root fresh weight under Cd and controlled conations compared to the no application conditions, while the increase in shoot fresh weight by applying 25 mg L^-1^ was negligible as compared to no ZnO-NPs application (**Figures 2C, D**). Moreover, the interaction between Cd stress and nanoparticle application for shoot and root fresh weight was not significant (**Table 2**). Shoot and root dry weight was significantly decreased by Cd stress compared to control. ZnO-NPs of 50 mg L^-1^ increased the shoot and root dry weight under the Cd and controlled conditions compared with no application. Shoot dry was higher under the higher dosage of nanoparticles under Cd stress, while the increased root was noted under the controlled condition compared to Cd stress with or without spraying nanoparticles (**Figures 2E, F**). Leaf area was not affected by Cd stress, while ZnO-NPs significantly increased leaf area. Higher leaf area was noted in controlled conditions compared to Cd stress under both dosages of nanoparticles (**Figure 2G**). Leaf area was highest in controlled conditions when 50 mg L^-1^ of ZnO-NPs were applied, while the interaction between Cd and nanoparticles for leaf area was not significant (**Table 2**).

**Table 2:** Analysis of variance (ANOVA) of data showing the differences in values of shoot and root length, shoot and root fresh weight, shoot and root dry weight, root dry weight, leaf area, chlorophyll a, b, total chlorophyll, and carotenoid contents of maize seedling by application of ZnO-NPs and Cd stress conditions.

Treatments	Shoot length (cm)	Root length (cm)	Shoot fresh weight (g)	Root fresh weight (g)	Shoot dry weight (g)	Root dry weight (g)	Leaf area (cm^2^)	Chl *a* (mg/g FW)	Chl *b* (mg/g FW)	Total Chl (mg/g FW)	Carotenoid (mg/g FW)
ANOVA											
Cd-Stress (A)	**	*	NS	NS	**	***	NS	*	**	**	*
ZnO-NPs (B)	***	**	**	***	**	*	***	**	**	***	*
A×B	*	NS	NS	NS	*	NS	NS	**	*	*	*

*, **, and *** show significant differences at probability levels of 0.05, 0.01, and 0.001, respectively. NS, not significant.

### 3.3 Photosynthetic pigments

**Figure 3:**
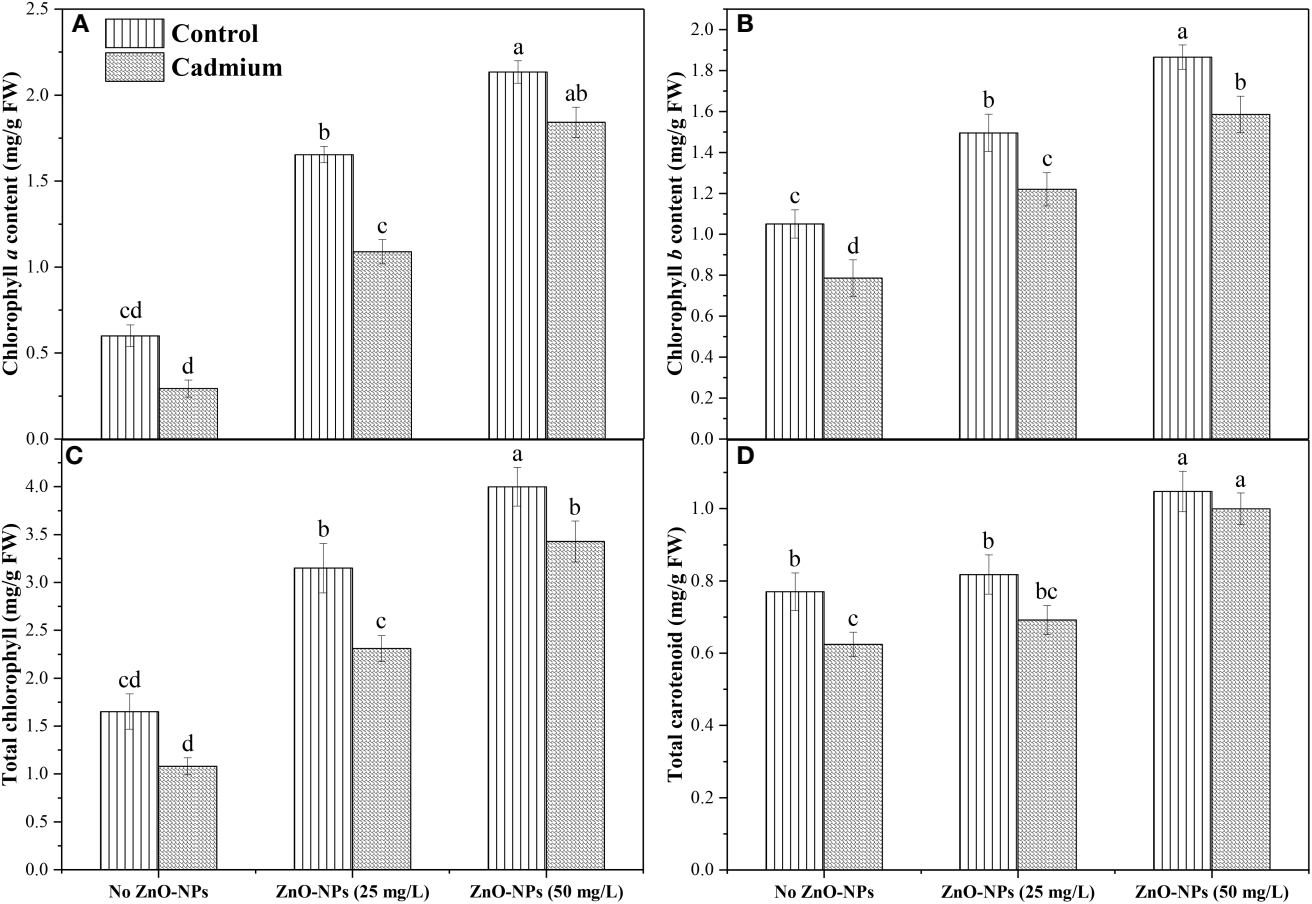
ZnO-NPs influence on **(A)** Chl *a*; **(B)** Chl *b*; **(C)** total chlorophyll; **(D)** total carotenoids (mg/g FW) at vegetative stage under cadmium stress and controlled condition. Three-way ANOVA was performed to evaluate the results. Bars with lowercase letters are statistically different at p< 0.05 by the LSD test.

Data analysis for Chl *a* and total chlorophyll exhibited a non-significant difference between control and Cd stress with no application of nanoparticle. Zinc-oxide nanoparticle dosages significantly increased total Chl and Chl *a* in controlled conditions as well as under Cd conditions. The highest Chl *a* and total Chl was observed when 50 mg L^-1^ of ZnO-NPs was applied (**Figures 3A, C**). Cd stress significantly decreased Chl *b* and carotenoid contents compared to control when no nanoparticles were applied. Application of ZnO-NPs in both dosages significantly increased Chl *b* under the controlled and Cd stress conditions. In contrast, carotenoid contents were significantly increased with 50 mg L^-1^ of ZnO-NPs application under the controlled and Cd stress conditions compared to no ZnO-NPs. 25 mg L^-1^ of nanoparticles also increased carotenoid contents under controlled and Cd stress, but this increase in carotenoid contents under the Cd stress was not statistically different with no nanoparticle’s application (**Figures 3B, D**). A significant interaction was observed between the two factors in Chl *a*, Chl *b*, total chlorophyll, and carotenoid contents (**Table 2**).

### 3.4 Metabolite accumulation

**Figure 4:**
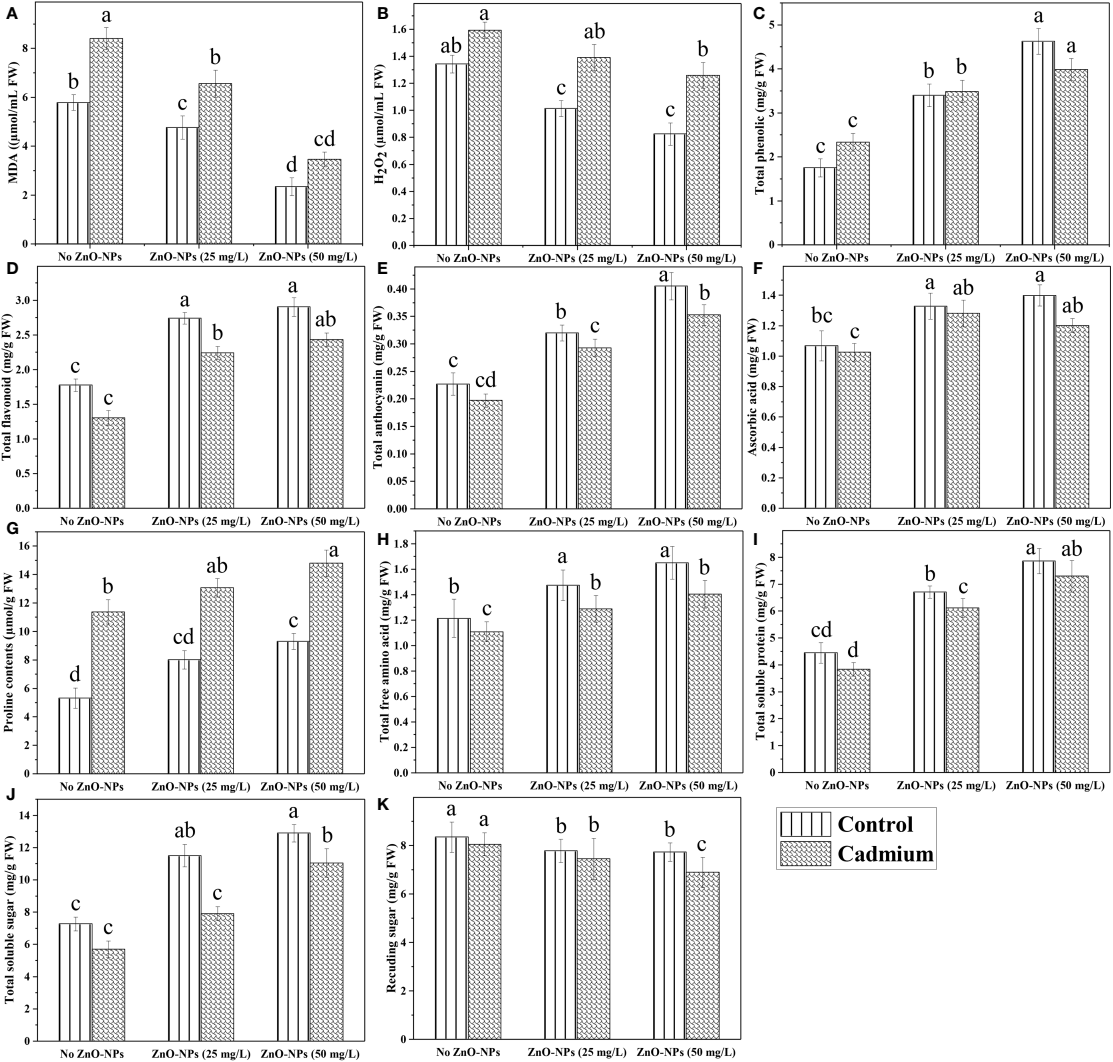
ZnO-NPs influence on endogenous contents of maize at vegetative stage under cadmium stress **(A–K)**. Three-way ANOVA was performed to evaluate the results. Bars with lowercase letters are statistically different at p< 0.05 by the LSD test.

Data analysis for malondialdehyde (MDA) showed a highly significant difference in Cd stress and ZnO-NPs. Cd stress significantly increased MDA contents with or without spraying ZnO-NPs. Zinc oxide nanoparticles in both dosages significantly decreased MDA contents compared to no ZnO-NPs. The lowest MDA contents were noticed with a higher dosage of nanoparticles, while these contents were higher when the nanoparticles dosage was decreased. Although MDA contents were decreased by applying ZnO-NPs compared with no application of nanoparticles, these were still higher compared to the control condition (**Figure 4A**). Similarly, H_2_O_2_ was also significantly increased in Cd-fed plants with or without nanoparticle application. Application of ZnO-NPs decreased H_2_O_2_ in control plants as compared to Cd-stressed plants, but its application had no significant effect on Cd-stressed plants (**Figure 4B**). A highly significant difference was detected in the effect of Cd stress on the proline contents of maize plants. Proline was significantly increased in Cd-fed plants with or without ZnO-NPs application. Although proline contents were increased in Cd-fed and controlled plants, that increase in proline contents was not statistically different. This means that the application of ZnO-NPs had a non-significant effect on proline contents (**Figure 4G**). Cd stress had a reducing effect on other metabolites such as phenolics, total flavonoids, anthocyanin, ascorbic acid, total soluble proteins, soluble sugar, reducing sugar contents as compared to controlled plants with no ZnO-NPs application, but that reduction in metabolic contents was not different statistically (**Table 3**). Application of ZnO-NPs in both dosages significantly increased above mentioned contents compared with no application of nanoparticles, but the difference between the effect of two different dosages of nanoparticles on most metabolites was non-significant. Such as total flavonoid, ascorbic acid and total free amino acids were increased by applying nanoparticles, but the difference between the two dosages was not significant (**Figure 4**).

**Table 3:** Analysis of variance (ANOVA) of data showing the differences in values of MDA, H_2_O_2_, total phenolic, total flavonoid, anthocyanin, ascorbic acid, proline contents, total free amino acid, total protein content, total soluble sugar, and reducing sugar of maize seedling by application of ZnO-NPs and Cd stress conditions.

Treatments	Malondialdehyde (µmol/mL FW)	Hydrogen peroxide (µmol/mL FW)	Total phenolic (mg/g FW)	Total flavonoid (mg/g FW)	Anthocyanin (mg/g FW)	Ascorbic acid (mg/g FW)	Proline contents (µmol/g FW)	Total free amino acid (mg/g FW)	Total soluble protein (mg/g FW)	Total soluble sugar (mg/g FW)	Reducing sugar (mg/g FW)
ANOVA											
Cd-Stress (A)	*	**	NS	**	**	*	***	*	*	**	*
ZnO-NPs (B)	***	*	***	***	**	NS	NS	**	**	**	***
A×B	**	**	NS	*	**	NS	NS	*	*	*	*

*, **, and *** show significant differences at probability levels of 0.05, 0.01, and 0.001, respectively. NS, not significant.

### 3.5 Enzymatic antioxidants

**Figure 5:**
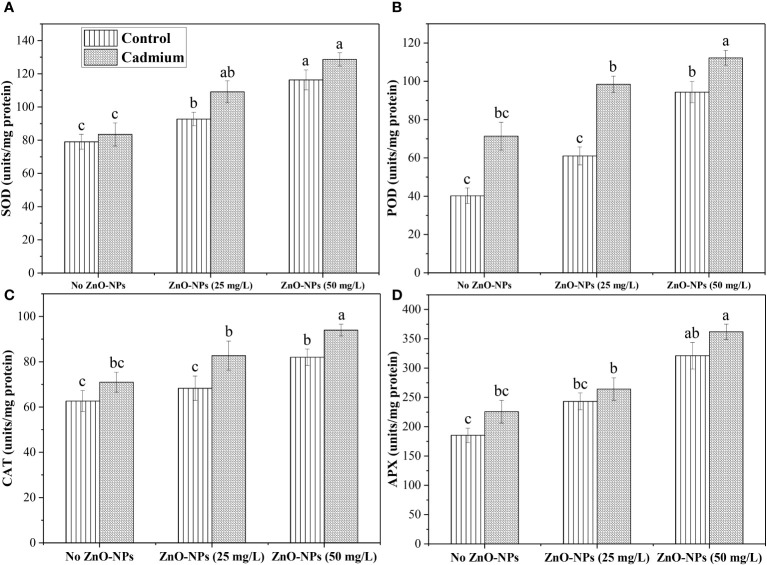
ZnO-NPs influence on antioxidants **(A)** Superoxide dismutase (SOD); **(B)** peroxidase (POD); **(C)** catalase (CAT); **(D)** ascorbate peroxidase (APX) (unit/mg protein) at vegetative stage under cadmium stress. Three-way ANOVA was performed to evaluate the results. Bars with lowercase letters are statistically different at p< 0.05 by the LSD test.

The SOD, POD, CAT, and APX data analysis expressed a non-significant difference between control and Cd-fed plants under no ZnO-NPs application. However, an increase in antioxidant activity was noticed in Cd-fed plants. ZnO-NPs dosages of 25 and 50 mg L^-1^ significantly increased SOD under controlled and Cd stress conditions compared to the plants without treatment of ZnO-NPs. There was no significant effect was noticed between 25 mg L^-1^ of ZnO-NPs and no nanoparticle treatment on POD, CAT and APX. A dosage of 50 mg L^-1^ increased these metabolites under Cd and controlled conditions. Overall, these antioxidants were noticed in higher concentrations under the Cd-fed plants compared to the control (**Figure 5**).

## 4 Discussion

In the present research, foliar application of ZnO-NPs in various concentrations alleviated the Cd stress and enhanced plant growth. We believed that NPs induced morpho-physiological changes in plant growth, root and shoot length, dry mass, primary metabolites, and antioxidant system depending on size, reactivity, chemical composition, surface contact, and dosage (Basit et al., 2023b). Moreover, Zn plays a vital role in maintaining and protecting cell membrane structure and is also used for abiotic stress tolerance, cell elongation, protein synthesis, and membrane functions (Ajouri et al., 2004). Raliya et al. (2015) revealed that treatment of ZnO-NPs with *Solanum lycopersicum* seeds resulted in increased root length, shoot length, and other growth attributes. *Vigna radiata* and *Cicer arietinum* roots, shoots and biomass were promoted by applying ZnO-NPs on the seedling stage. Researchers are trying hard to improve crop efficiency by modifying the physiological and biochemical traits (Mahajan et al., 2011). The outcomes presented that exogenously applied ZnO-NPs accelerated maize biomass and growth. Plant height and biomass are the two basic indications of abiotic stress (Rizwan et al., 2019b). Higher Cd levels in maize are related to the control plants’ reduced biomass. The resemblance between the non-essential element Cd and the necessary nutrient Zn increased the zinc relevance in the soil-plant relationship (Rizwan et al., 2019a). Due to the incompatible actions of these metals on each other, a sufficient amount of Zn in the soil may interact with Cd and limit plant buildup (Huang et al., 2019; Hussain et al., 2022b; Hussain et al., 2022c).

The photosynthetic machinery is regarded as the chief indicator of heavy metal-induced poisonousness in plants (Faseela et al., 2020). Similarly, heavy metal stress altered the photochemistry of chlorophyll (Chl *a*, Chl *b*) and carotenoid contents for light harvesting and, as a result, the primary component of the photosynthetic process (Ulhassan et al., 2019; Ulhassan et al., 2022b). Similar findings revealed in our study, chl *b* and carotenoid contents lessened (35% and 19%) under Cd stress compared to control plants. Foliar application of ZnO-NPs (25 and 50 mg L^-1^) improved the Chl *a*, *b*, total chlorophyll in controlled conditions. Total Chl and carotenoids were increased by ZnO-NPs (50 mg L^-1^) in cadmium-fed plants (**Figure 3**). Heavy metal stress like Cd in several plants produces excessive ROS, damaging cells. (Burman et al., 2013) observed that ZnO-NPs accelerated chickpea root and shoot growth. Some chickpea genotypes have also been observed to exhibit a change in the root: shoot ratio due to zinc supplementation. However, in rice cultivars, plant heights, RL, SL, FW, DW, and leaf area were massively reduced through Cd stress (Faizan et al., 2021). Likewise, in our recent study, ZnO-NPs reduced the heavy metal stress (Cd) in maize crops and improved the morphological parameters. Cadmium toxicity produces oxidative stress and reactive oxygen species, which alters enzymatic activity due to the blocking of proteins, histidyl, carboxy, and thiol. In radish (*Raphanus sativus* L.), metal toxicity often arises in excessive ROS, which oxidizes proteins, lipids, DNA, and other biological components (Noman et al., 2018). Compared to the corresponding control, the elevated levels of MDA and H_2_O_2_ in the leaves and roots were reduced at 100 mg L^-1^ of ZnO-NPs (Rizwan et al., 2019a). Previous research found that adding ZnO-NPs into the Cd-stressed tomato plants significantly enhanced proline buildup. ZnO-NPs may increase proline by mediating proline biosynthesis gene expression (Faizan et al., 2021). Similar findings were seen in our research, including elevated levels of MDA, H_2_O_2_, and proline also altered the antioxidant enzyme activity (**Figure 4**). Under Cd stress, there was an increase in MDA (45%), H_2_O_2_ (18.6%) and proline (114%) levels, ZnO-NPs (25 and 50 mg L^-1^) foliar spray increased proline (15% and 30.03%) but lessened MDA and H_2_O_2_ (21% and 59%, 13% and 21%).

In a recent study, the total soluble protein was decreased because of the heavy metal administered to *Solanum lycopersicum* through the soil. This might be caused by a decrease in the production of protein macromolecules under this stress and an increase in the rate of protein denaturation caused by protease activity. Furthermore, stressed and non-stressed plants have higher protein contents after receiving treatment with ZnO-NPs as an exogenous spray and epibrassinolide as a root dipping solution (Faizan et al., 2021). Zinc, as a micronutrient, has a vital role in plant development and its deficiency causes incompetence in the enzymatic system and physiological stress and also crucial for the production of tryptophan. Correspondingly, our study showed that total soluble protein content decreased (14%) under Cd stress in maize while increased (51% and 77%) by the exogenous spray of growth regulator ZnO-NPs (25 and 50 mg L^-1^). In our current findings, ascorbic acid content decreased (2.25%) with Cd stress but increased (24.40% and 31.02%) with foliar application of ZnO-NPs (25 and 50 mg L^-1^) (**Figure 4F**). These outcomes correspond with earlier studies on tomato plants. The concentration of ascorbic acid increased in both tomato cultivars, which was more significant under higher concentrations of heavy metal under stress conditions (Hussain et al., 2017). High Cd concentrations substantially elevated metabolites such as anthocyanin content in maize (Qutab et al., 2017).

Similarly, according to (Thiruvengadam and Chung, 2015), turnips possess higher anthocyanin levels under Cd stress. Likewise, Cd stress increased phenolic’ levels while reducing the flavonoid content (Thiruvengadam and Chung, 2015). In our findings, Cd stress dropped the total phenolic, flavonoids, and anthocyanin contents, while ZnO-NPs (25 and 50 mg L^-1^) improved (49.26% and 71%), (72.02% and 86.47%), (41% and 78.41%) these secondary metabolites by reducing the Cd stress effect, respectively (**Figures 4D, E**). The impact of various Cd concentrations on two *Pisum sativum* genotypes (AG-10 and AP-3) was assessed by Sager et al. (2020). Cd stress lessened the amount of sugar in both genotypes. Our experiment revealed the same results (**Figures 4J, K**).

Additionally, the effects of Zn applied topically to two maize cultivars under metal stress cause a decreased stress and a rise in total free amino acid. Our present study revealed similar findings. The total protein content and total free amino acid concentration decreased under Cd stress and increased (59.42%, 90.18% and 16.30%, 27%) with the efficacy of ZnO-NPs (25 and 50 mg L^-1^) (**Figures 4J, H**). Earlier research has shown that ZnO-NPs strengthened the antioxidant system and increased SOD, POD, CAT, and APX activities (García-López et al., 2019; Ulhassan et al., 2022a). Adrees et al. (2021) revealed that when ZnO-NPs were applied, Cd stress was reduced by 26% and 81% in normal moisture, respectively. ZnO-NPs, when applied topically to leaves, improved plant growth by boosting antioxidant defenses and decreasing oxidative stress while also increasing SOD, POD activities. Likewise, our findings indicated that the overall uptake of Cd by various plant parts was more significant under the given treatment than in controlled plants. A foliar spray of ZnO-NPs improved the antioxidant enzymes (POD, SOD, APX, and CAT) concentrations. SOD and POD were increased by spraying ZnO-NPs (25 and 50 mg L^-1^) under Cd stress (**Figures 5A, B**). The activity of CAT and APX was enhanced in cadmium-fed plants and increased by spraying ZnO-NPs (25 and 50 L^-1^) in the control and stressed plant (**Figures 5C, D**). However, the mechanism and the reasons for the enhanced antioxidant system and maintained metabolite accumulation in the plants exposed to NPs have not been fully studied. There is a need to further investigate the mechanisms of NPs mediated changes for sustainable agriculture practices.

## 5 Conclusion

In the current study, two Cd levels (0, 0.6 mM) and zinc oxide nanoparticles (25 and 50 mg L^-1^) were applied to the maize seedling to investigate the changes in plant growth attributes, total chlorophyll, carotenoid, primary metabolites, and antioxidant system.

In conclusion, Cd stress decreased shoot and root dry weight compared to controlled conditions with no zinc oxide application, while ZnO-NPs enhanced and improved the morphological and physio-chemical attributes. Cd toxicity reduced Chl *b*, carotenoid contents and metabolic activity compared to control with no foliar application of ZnO-NPs. Zinc-oxide nanoparticle application enhanced plant growth by maintaining primary and secondary metabolites and antioxidant systems. Foliar application of ZnO-NPs in different dosages (25 and 50 mg L^-1^) increased antioxidants (SOD, POD, CAT, APX), photosynthetic pigments, metabolites including total flavonoid, total phenolic, total anthocyanin, ascorbic acid, proline contents, total free amino acid, total soluble proteins, and soluble sugar compared with no foliar application conditions under the controlled and Cd stress. At the same time, it caused a reduction in MDA, H_2_O_2_, and reducing sugar in the cadmium-fed plants. Current research proved that Cd stress reduces the progress of growth and development in plants. It is recommended to apply ZnO-NPs to improve the capacity of tolerance in maize plants under Cd stress. The hazardous effects of Cd stress can be lessened with the efficacy of ZnO-NPs. Maize showed better growth when ZnO-NPs (50 mg L^-1^) were applied exogenously under Cd stress. Overall, the foliar efficacy of ZnO-NPs enhanced the tolerance capacity of the maize plant.

## Data availability statement

The raw data supporting the conclusions of this article will be made available by the authors, without undue reservation.

## Author contributions

MH: Formal Analysis, Investigation, Methodology, Writing – original draft, Writing – review & editing. RK: Conceptualization, Methodology, Visualization, Writing – review & editing. SH: Data curation, Software, Writing – review & editing. CS: Formal Analysis, Investigation, Writing – review & editing. GW: Validation, Visualization, Writing – review & editing. NR: Investigation, Validation, Writing – review & editing. WS: Resources, Validation, Writing – review & editing. YL: Writing – review & editing, Funding acquisition, Supervision, Validation, Visualization.
